# The evolving landscape of master of public health (MPH) programs in India: a desk review

**DOI:** 10.3389/fpubh.2026.1865070

**Published:** 2026-07-16

**Authors:** Shanti Dahal, Tapaswi Puwar, Sanjay Zodpey

**Affiliations:** 1 Public Health Foundation of India, Gurugram, Haryana, India; 2 Indian Institute of Public Health-Gandhinagar, Lekawada, Gujarat, India

**Keywords:** accreditation, competency-based education, health workforce, master of public health, public health education

## Abstract

**Introduction:**

India’s evolving public health challenges emphasize the need for a competent, multidisciplinary public health workforce, emphasizing the importance of strengthening public health education. Since 1995, the Master of Public Health (MPH) programs in India have expanded rapidly; however, in the absence of a regulatory body, consolidated details regarding these programs remain not readily available. This study aimed at landscaping MPH programs in India and understand its key components in terms of geographical distribution, admission criteria, fee structure, electives, program delivery modes, internships, and dissertation requirements.

**Methods:**

To compile a comprehensive overview of MPH programs across India a multi-step desk review was undertaken.

**Results:**

The study identified 177 institutions offering MPH programs with intake capacity of 4,228 seats (data available from 136 institutes) across 23 states and three Union Territories. Variations were seen across institutions in program eligibility criteria, admission processes, program electives, program fees, credit requirements, the structure and duration of internships and dissertations.

**Discussion:**

The expansion of MPH programs during last three decades, highlights growing recognition of public health as a multidisciplinary field; however, the variations in program offerings and admission criteria highlight the lack of standardized structure in MPH programs across India. Introducing a standardized admission test for MPH in India, multi-disciplinarity in the program admission criteria and eligibility, developing a competency-based MPH curriculum aligning with the evolving employment market, and a national accreditation body could significantly strengthen MPH education, leading to workforce preparedness to address India’s emerging public health priorities.

## Introduction

Globally, the increasing complexity of public health challenges has emphasized the importance of a well-developed public health workforce which is equipped to tackle the challenges of complex and evolving public health needs. Thus, it is crucial to develop a robust public health workforce that can improve the health outcomes by contributing effectively towards delivery of quality health services (1).

The focus on public health education in India began as early as the 1940s with the establishment of Bhore Committee (2, 3). The Committee, highlighted the gaps in medical education curriculum and stressed the importance of training the doctors as social physicians by including preventive medicine and public health in the curriculum (2). Further, in 1999, the ‘Calcutta Declaration on Public Health’, outlined strategies to strengthen public health education and build a stronger public health workforce in the South East Asia region including India (4, 5). India’s complex public health challenges due to its large and diverse population, ranging from socio-economic inequalities, growing burden of communicable and non-communicable diseases, rapid urbanization, environmental health and climate change etc. (6) highlights the need for a strong public health education to provide quality healthcare for its large population (7). The need for new postgraduate courses and institutes for public health and hospital management, need to update health curricula, build training centers, and form partnerships to create skilled public health professionals were recommended by, the High Level Expert Group on Universal Health Coverage in 2012 (8, 9). Furthermore, National Health Policy (NHP) 2017 called for creating a Public Health Management Cadre (PHMC) to improve India’s health system (10), aiming to attract young professionals from different fields to meet workforce gaps (11), leading to launch of Public Health Management Cadre Policy in 2022 for implementation (12).

Public health education in India was traditionally offered through medical colleges by way of MBBS (Bachelor of Medicine and Bachelor of Surgery) and MD (Doctor of Medicine) in Community Medicine or Public Health programs (2). However, these trainings were largely focused on clinical aspects and less towards health systems and community-level initiatives, which provided limited exposure to the multidisciplinary nature of public health. Gradually over the years, there was a conscious shift towards opening the schools of public health in India to deliver health system specific Master of Public Health (MPH) program. The first school of public health was started in the south state of India- Kerala, by Mahatma Gandhi University, Kottayam in 1995, and over the last 3 decades, there was an expansion in the number of institutes delivering MPH programs, to 116, in the year 2024 (13). However, due to the absence of a formal body or council for regulating public health education in the country, till date, there is limited information available on evolution, development and issues related to MPH programs in India (8). Furthermore, in the absence of a regulatory body for MPH education in India, the data about MPH programs is not readily available on a single platform as well (14).

With this background, the present study explores the landscape of MPH programs in India. The study looks into the key aspects of the program- program eligibility, admission criteria and process, geographical distribution of the institutes, modes of program delivery, program fee, electives, specializations, and components like internships and dissertations. The study also examines the multidisciplinarity of these programs.

## Methods

A multi-step process was undertaken to identify institutes offering MPH programs in India.

As a first step, various publications on public health education in India were reviewed to identify institutes offering Master Level Public Health programs in India, sourced through Google Scholar, Research Gate, PubMed.

The next step involved using specific keywords through google search to collate the information available in the public domain. Keywords used for the search were MPH programs in India, Master’s level programs in public health in India, Master of Public Health at India, Master in Public Health offered in India, Public Health Institutes in India, Indian MPH programs, Postgraduate programs in Public Health. Additional data were compiled from National Medical Commission (NMC), University Grants Commission (UGC), educational portals, newspapers, and social media platforms.

As a third step, the data was validated through phone calls to the institutes, email correspondence, and scanning institutional websites for the course handbook to get insights into course details. The fourth step involved filtering out only those institutes/Universities offering MPH programs and MA/MSc in Public Health. The final step involved filtering out institutions that were offering only MPH programs.

Finally, the list was categorized by specializations offered and mode of delivery of the MPH program. Further categorization was done for zonal distribution of the institutes as per the Government of India identified geographic zones. The list was further narrowed down to public and private institutes and medical and non-medical institutes. This multi-step methodological approach helped to compile a comprehensive list of zone-wise distribution of the institutes offering MPH programs in India.

Only MPH programs offered as full-time/part-time programs or Integrated MPH programs in different modes were included in the final analysis. The duration of the program ranges from 1 to 3 years. The study excluded institutes offering Bachelor level programs in Public Health, MD (Community Medicine), MD (Preventive and Social Medicine), Post graduate diploma programs in Public Health.

Ethical approval for the study was taken from the Institutional Ethics Committee, Indian Institute of Public Health, Gandhinagar University.

**Figure 1:**
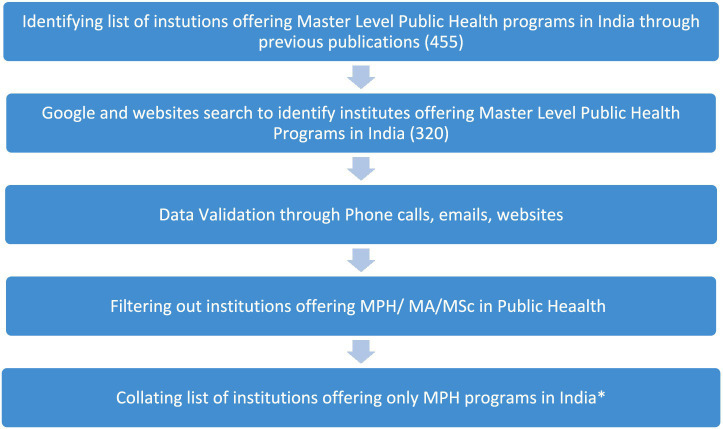
Process of finalizing institutes offering MPH programs in India.* also includes integrated MPH programs.

Figure 1 shows the steps followed.

## Results

### Number of MPH institutes, intake capacity, geographical distribution and presence of separate departments or schools for MPH

A total of 775 institutes offering Master level programs in public health were identified through desk review which was further filtered out to remove duplicates and unrelated programs and identified the list of 177 institutes offering MPH programs in India in year 2025. This also includes institutes offering Integrated BSc/BPH and MPH programs. The duration of program ranges from 1 to 3 years.

Through desk review, the intake capacity data for 136 institutes offering MPH programs was collected. The data shows a total of 4,228 seats, including 30 seats for integrated MPH programs. Additionally, 13 institutes offer MSc and MA programs in Public Health, with six of these institutes providing both MPH and MSc/MA courses. The total intake capacity for MA and MSc Public Health programs is 226 seats.

The distribution of MPH institutions according to zones and their public or private status is given in Table 1.

**Table 1:** Zonal distribution and distribution of institutes of MPH institutes in India, per their management (According to the States Reorganization Act of 1956, India established six zonal councils, namely Central, East, North, North East, South, and West).

Zone	Number of Institutes	Public Institutes (MPH)	Private Institutes (MPH)	MPH including Integrated MPH	MSc/MA etc.	Integrated BPH/BSc and MPH
North	46	16	28	44	4^*^	
East	30	6	23	29	3^*^	1^^^
West	31	13	17	30	2^*^	
South	42	8	32	40	3^*^	1^^^^
North-east	5	–	5	5		
Central	31	6	23	29	1	
Total	185	49	128	177	13	2

Public institutes are government institutes and private institutes are self-funded institutes.

13 Institutes offering Programs with title as MA/MSc in Public Health/MSc Epidemiology and Public Health etc.

^*^Six institutes are offering MPH as well as MSc/MA courses; 2 in East, 2 in North, 1 in South, 1 in West.

^^^Included in MPH.

^^^^One institute is offering MPH and also BSc and MPH (Integrated). Included in MPH.

Integrated BPH/BSc and MPH are 5 year duration programs leading to final degree of Master of Public Health.

For the study we only considered MA/MSc programs with inclusion of public health in the program nomenclature.

It was found that, in India, 1-year MPH programs and Integrated BSc and MPH programs are also introduced by few institutes. In 2025, one private institute offered BSc–MPH (Integrated) of 5 years’ duration, with the option of multiple entry and exit. Apart from this, one government institute started India’s first dual degree program in public health- Integrated Program in Public Health (BPH + MPH) in July 2025.

One institute offered the unique 1-year hybrid Master of Public Health specialization in Community Health (MPH-CH). The program offers the flexibility of lateral entry to candidates. One of the institutes recognized as Institute of Eminence transformed its 2 years MPH program to 1-year program in 2025, and the program is open for fresh graduates.

Three years MPH program is offered by two institutes in India- one at East (MPH- Epidemiology) and another at South India (MPH honors).

Out of 177 institutions, we could identify 118 [66%] institutes having a separate school or centre or department for offering MPH programs. Although most of the department names represent public health, few institutes are running the MPH program under departments such as Allied Health Sciences, Commerce and Management, Arts etc.

Eleven institutes earlier reported in a recently published study in 2024 (13) have discontinued their programs.

### Geographical distribution of MPH programs

At present in India, MPH is being offered in 23 states and 3 Union Territories (UT) of Delhi, Chandigarh and Puducherry. Maharashtra has highest number of institutes (15) followed by Karnataka (16), Odisha (17) and Rajasthan (18). In Odisha, Utkal University has affiliated 10 institutes for MPH education. States of Himachal Pradesh, Jharkhand, Chattisgarh, Andhra Pradesh and North east states have the lowest number of institutes (1–2 in each). Interestingly, states of Mizoram, Arunachal Pradesh, Bihar, Assam and Goa do not have any institute offering MPH program. Among the Union Territories only The Government of National Capital Territory (NCT) of Delhi, Chandigarh and Puducherry have institutes offering MPH programs.

**Figure 2:**
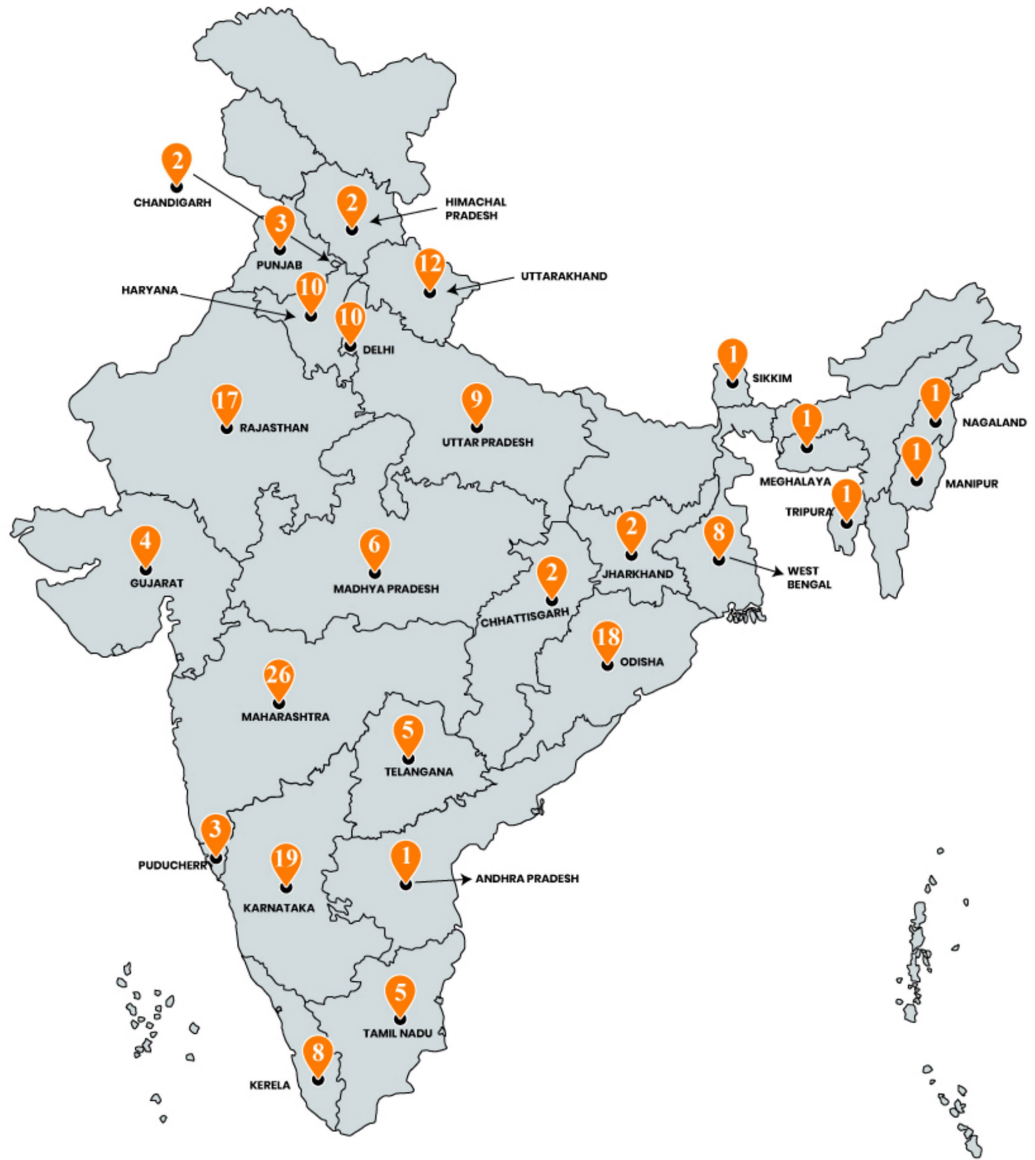
Number of MPH institutes (State Wise)In India, there are 28 states and 8 Union territories. In India, Union Territory (UT) is an administrative region administered by the President of India through an Administrator or Lieutenant Governor appointed by the President.

Figure 2 gives details of number of MPH institutes in each state.

### Program nomenclature and mode of offering

MPH program is offered with various nomenclatures. While most of the institutes [152, 86%] name it as MPH, some institutes [29, 16%] offer it by the name of MPH with specialization. Ten institutes are offering both MPH and MPH with specialization. Three institutes [approx. 1%] are offering MPH both in on campus as well as Hybrid mode. Recently few institutes [7, 4%] have started offering Executive MPH programs as well. Two institutes are offering MPH in part time mode, while nine are offering also in online and hybrid mode. Most of the institutes offer MPH in on campus mode.

Interestingly, within MPH the full form also varies from Master in Public Health, Master of Public Health, Masters in Public Health, Masters of Public Health. One institute offering Master in Science in Public Health and another one offering Master of Public Health Sciences calls the program as MPH and MPHS.

### Eligibility criteria for MPH programs

Out of 177 institutes, program brochures of 168 were available, which was scanned to understand the multidisciplinarity in MPH admissions eligibility criteria. 50 institutes [30%] reported enrolling any graduate for MPH program, while the rest give preferences to medical graduates; Bachelor of Dental Surgery (BDS); Ayurveda, Yoga & Naturopathy, Unani, Siddha, and Homeopathy (AYUSH); Nursing; Social Sciences; Allied and Health Sciences; Life Sciences graduate.

Interestingly commerce [7, 4%], engineering [19, 11%] and law [29, 17%] were specifically included in eligibility criteria of few institutes. Twenty-nine institutes [17%] preferred 1–3 years’ work experience.

### Admission process for MPH programs

The admission process for MPH programs was analyzed through scanning the website, admission brochure and telephonic enquiry. Out of 177 institutes, the data was captured for 145 institutes. The most common process followed are Entrance Examinations [82 institutes, 57%], Academic Merit [22 institutes, 15%], Personal Interviews (17 institutes, 12%). However most of the institutes use a combination of entrance examination, interviews, Statement of Purpose (SOP), work experience and academic merit for admission in the program. It is interesting to note that 22 institutes [15%] mentioned that they give direct admission to the students on first come first serve basis.

Since there is no standard national level entrance test for MPH admission, institutes follow different tests at institutional level.

### MPH program fee structure and credits

The fee structure for MPH programs was analyzed through scanning the website, course handbook and telephonic enquiry. Out of 177 institutes, the data regarding fee structure was captured for 166 institutes. The fee structure of the program also varies across institutes. The highest total program fee for MPH program is INR 19,52,723/−(USD 20,671.41) by a private institute, where the MPH program is run in collaboration with a foreign University and lowest total program fee is INR 707/−(USD 7.48) by a public institute. The MPH fee data also includes fee for integrated MPH programs offered by two institutes.

For MA/MSc programs the highest total program, fee reported is INR 140,000/−(USD 1,482.03) and Lowest total program fee is INR 23,685/−(USD 250.73).

Out of 177 institutes, the data regarding credits was captured from course handbook of 77 institutes. A course credit is academic unit of value assigned to a specific class which measures and reflects the amount of learning in a course and helps track progress towards a qualification (19). The MPH course credit also varies across institutes, with lowest credit of 40 and highest credit of 197. For the Integrated MPH programs, the highest credit is 240 and lowest is 220.

### MPH program offered in medical institutes/colleges

MPH program is also offered in some Medical Colleges. Currently, among the 177 institutes offering MPH programs in India, 43 are medical institutes [24%], which also includes one institute offering Integrated Program in Public Health (BPH + MPH). Maharashtra has the highest number of medical colleges offering MPH programs (14 colleges), which includes nine government medical colleges offering 2 years MPH (Nutrition) program. This is followed by Karnataka (six colleges), Kerala (four colleges) and Rajasthan and Uttarakhand (three colleges each).

### Program electives

We scanned the course handbook of institutes available on websites and also details shared by the course coordinator to understand the electives being offered in MPH. Program electives are the optional specialized courses being offered in MPH which a student can select based upon his interest areas to meet the program learning objectives (20).

Out of 177 institutes, the data was captured for seventy-five institutes who reported that they offer electives in MPH. Course coordinators from seven institutes did not share the list of electives but specifically mentioned that electives are offered. While typically the electives are offered in 3rd and 4th semester, few institutes mentioned that the electives are offered from 1st semester onwards in each semester. Some are also offering it from 2nd semester onwards.

### Internship and dissertation

Eighty-five schools [48%] out of 177 reported they have dedicated internship programs in 3rd or 4th semester of the MPH. Few of these (17 institutes) also have internship component at the end of 2nd semester with few of them in 2nd, 3rd and 4th semester and one institute reported 1-hour weekly internship.

One hundred and four institutes [59%] out of 177 mentioned that they have dissertations in MPH. While most of the dissertations are of 6 months in 4th semester, some institutes also offer dissertation from 3rd semester onwards. Two institutes mentioned that they have a capstone project in 2nd year of the program. One institute mentioned that it has 2 months’ dissertation in 4th semester. The dissertation ranges from 3 to 6 months.

## Discussion

India’s complex public health challenges emphasize the need for a well-developed public health workforce to effectively deal with these challenges and implement public health policies. The growing importance of public health both at national and global level has reinforced the need for a well-developed and strong health system for accomplishing the Sustainable Development Goals (SDGs) (18).

There is a mismatch between demand and supply of public health professionals. However, these skilled professionals are required across the entire health system (17). By the year 2030, India would need approximately 80 million health workers (16). The need for public health professionals would be additional to it. In this context, MPH programs play an important role by creating a flow of public health professionals in the system by advancing public health education. These programs equip professionals with the knowledge and skills required to work in public health.

Over the years, the MPH programs have evolved with an increase in the number of institutions offering MPH programs in India. Evolution of MPH programs in India is given in a snapshot, in Table 2 (8, 13, 21–23).

**Table 2:** Scaling up of MPH programs in India.

Year	No of Institutes offering MPH programs	Intake capacity/seats
1995	1	20
1997	2	35
2005	4	75
2010	23	573
2016	44	1,190
2021	105	1,722
2024	116	Data not available
2025^*^	177	4,228 (data regarding intake capacity available from 136 institutes)

^*^Current status of institutes offering MPH programs in India, as per the present study.

Over the past three decades, in India, the landscape of MPH programs has expanded considerably, driven by the country’s growing recognition of public health as a distinct and multidisciplinary field (13). With the rapid development in the field of public health and emerging areas, there is also a need for a well-developed multidisciplinary public health workforce equipped with the skills to deal with the public health challenges of 21st century (24). This has led to introduction of electives and specialized MPH programs in India to prepare graduates for roles in research, policy-making, program implementation, global health and other areas of work. This expansion not only diversifies career pathways but also strengthens India’s capacity to respond to national and international health priorities.

Apart from specialized MPH programs, there is a need to evolve the admission process and eligibility criteria for MPH programs in India. The present study found that the eligibility criteria in MPH programs are still focused on giving preferences to medical, AYUSH, nursing, Dentistry, life sciences and social sciences backgrounds, with very limited options for students from areas like law, commerce, engineering etc. limiting the scope of multidisciplinarity in admissions. Since public health draws knowledge and integrates work from various disciplines (25), this calls for a need to evolve the eligibility criteria for MPH admissions in India with the institutes opting for any graduate as a standard benchmark for the eligibility.

Unlike other professional courses like Medical, Management, Engineering etc., in India, till date there is no standard national level entrance test for MPH admission, which brings challenge to the institutes as each following their own entrance test. This brings variation in selection of the students in terms of their knowledge and aptitude. It is thus recommended that a National Level Entrance Test for MPH admissions be introduced in India. The entrance test needs to be monitored by a central body, and results of this test can be accessed by MPH institutes across India by paying a nominal charge. The institutes can have autonomy in deciding the minimum score required to take admission in the program at their institute. This can result in bringing the best talent to the institute with a right mix of aptitude and knowledge for public health.

At present the MPH programs are offered under various departments and schools lacking a uniformity in the names. MPH program offered through an unrelated department or school may lose its context and also dilute the quality of education (13). Further inclusion of a uniform department or school/centre for offering MPH programs will also help prospective students navigate the institute website to identify and apply for the specific course they are searching. This will also help the institute increase visibility of their MPH program.

With the introduction of National Education Policy (NEP) 2020 and its mandate for competency based education (CBE), Institutes offering MPH programs in India should now focus more on aligning the MPH curriculum with the requirements of the evolving employment market. While the CBE has been mandated by NEP (2020) (15), it is partially being adopted in various institutes, largely because of the limited capacity and expertise. The NEP 2020 also allows, students of 4 year degree courses to complete their post-graduation in 1 year (15), thus the institutes have a scope of re-visiting the MPH curriculum in alignment with NEP 2020.

In 2017–18 The Ministry of Health and Family Welfare (MOHFW) released its first model curriculum for the MPH (26). The UGC had requested institutes offering MPH programs in India to adopt this curriculum. However, there is little evidence related to adaptation of this curriculum by the institutes (23), largely because of the absence of a national accrediting body for MPH education in India. The MPH model course curriculum recommends total 50 credits for the MPH program which includes one elective course (26), however our study figured that the course credits varies across institutions, with no standard structure being followed. Institutes should focus on standardizing their curriculum by offering the right mix of electives and core modules. The course credits should focus on offering electives in relevant areas to enable the students to acquire competencies in the core domain of work in public health and also developing the cross-cutting competencies among students. Some examples of cross cutting competencies would be leadership and management, collaborations and partnerships, communication skills, digital skills, decision making, analytical skills, systems thinking, community engagement etc. This would also mean re-adjusting the course credits from a fresh perspective. It is recommended that the MPH programs should have a mix of 2 electives in core areas of work and 1–2 electives in cross-cutting areas. Alternatively, the cross-cutting areas can be offered as a part of each module as a mandatory subject/topic. For example, a core area of health policy and management can have topics/assignments related to management, decision making, systems thinking etc. Inclusion of core and cross-cutting competencies in the MPH curriculum would ensure a match between educational and work level competencies and thus preparedness of students to meet the requirements of public health employment.

MPH program should have a component of mandatory internship and dissertation to be submitted at the end of fourth semester, which will help students develop practical skills in public health (26). However, the evidence available from the present study shows that the internship and dissertation are poorly structured across Institutes. The course handbook of institutes available many times do not specifically mention the details of internship and dissertation. It is recommended that the fourth semester of the MPH program should be dedicated towards a dissertation of 4 months followed by an internship of 2 months. This pattern of offering will help in enhancing the student placements towards the program completion. Employers are more likely to absorb the students in a full-time role after completion of the internship program, provided there is no long gap between internship and full time joining. Another area of improvement in terms of the course curriculum would be the focus on imparting community/field immersions apart from the internship and dissertation. Such experience help students gain practice based skills, helpful for securing employment.

India has a wide pool of public health professionals many of whom are lacking formal Master degree in Public Health, thus, it is recommended that the regular 2-year MPH programs offer the flexibility of lateral entry options, allowing candidates with prior work experience in public health with a prior postgraduate diploma in public health or related field, for direct admission into the second year of MPH. Similarly, the Integrated MPH (IMPH) program, recently introduced by two institutions in India, brings an innovative perspective in MPH education. These programs give students the scope of gaining an in-depth understanding of public health. These programs should focus on introducing a 1-year internship in the final year to help students develop employment-specific skills, aligning with the requirements of competency-based education, as mandated by NEP 2020. Inclusion of multiple entry and exit options in the program will offer flexibility to students and also increase the acceptance of the program. This system gives students the flexibility of learning by enabling them to exit and re-enter their studies while retaining their earned credits (15).

The Integrated MPH (IMPH) programs could help bridge the skill gap in terms of providing practice based training to students, as the mandatory 1-year internship in the final year will provide students exposure in both employment-specific skills and valuable hands-on experience, enhancing their career readiness. The students can be posted as “Public Health Trainee” in an organization, a position which is not present in India till date at government or private organizations. Under this model, fresh MPH graduates could spend their internship in rotational postings across different departments/projects (2–3 departments in 1 year), gaining exposure into different area of work. Upon successful completion of the internship and based on performance appraisals, trainees could either be absorbed into full-time positions or they can apply for roles in other organizations. This will be a win-win situation for institutes, students and employers. The institutes would see enhanced student placements which will also help increase their enrolment numbers for new batches, MPH students would enter the workforce with employment-specific competencies supporting their career, and employers would gain access to well-prepared, skilled professionals.

The study identified emerging trends in the delivery of MPH programs, including Executive MPH (EMPH), part-time, hybrid, and online MPH programs which are specifically designed to meet the needs of program participants, particularly the working professionals. The online programs do not align with the requirement of UGC under regular education. However, the introduction of EMPH, part time and hybrid MPH program formats is a welcome step for working professionals, as it provides the flexibility to pursue higher studies while continuing employment.

It is important to create a linkage between academia and the health system such that training and capacity-building efforts in MPH education in India are aligned with workforce priorities. A welcome step in this direction is the Network of Schools of Public Health in India (NSPHI), which has been recently established in India (27). This network brings together 13 leading schools of public health in India at a common platform, which collaboratively work for advancing public health education and standardization of MPH curriculum. This network could advocate for a national body, like a Public Health Education Council of India (PHECI), for accreditation of public health academic institutes, MPH admission entrance test in India and MPH programs.

## Conclusion

The study provides a comprehensive and updated landscape analysis of MPH programs in India, with details of intake capacity, program fee, mode of program offerings and launch of Integrated MPH programs in India, and discussing the variations in MPH programs offered in India, creating a consolidated resource for future research in public health education which was previously lacking. The recommendation of this study highlights the need for broader structural reforms in public health education along with critical gaps in MPH curriculum which is valuable for policymakers, academic institutions and employers to bring a health system connect in MPH education in India and standardize the curriculum which would further strengthen MPH education in India.

One of the limitations of this study is the desk review mainly captured MPH program specific information for the study. However, in the absence of a regulating body for MPH programs in India, details of some institutions may not have been captured in the study. There were variations in the number of institutes during final analysis, mainly due to the unavailability of complete data during desk review. Further, the details of institutions captured in the study need to be updated periodically as the landscape of MPH education in India is continually evolving. With the expansion of number of institutes offering, every year new institutions launch MPH programs and few institutions discontinue offering the programs.

Despite of these limitations, the study provides a comprehensive overview of the current landscape of institutions offering MPH programs in India and establishes a baseline and reference for future research on public health education in India.

## Data Availability

The raw data supporting the conclusions of this article will be made available by the authors, without undue reservation.
